# 3D-Stacked Multistage Inertial Microfluidic Chip for High-Throughput Enrichment of Circulating Tumor Cells

**DOI:** 10.34133/2022/9829287

**Published:** 2022-07-18

**Authors:** X. Xu, X. Huang, J. Sun, J. Chen, G. Wu, Y. Yao, N. Zhou, S. Wang, L. Sun

**Affiliations:** ^1^ Ministry of Education Key Laboratory of RF Circuits and Systems, Hangzhou Dianzi University, Hangzhou, 310018 Zhejiang, China; ^2^ Institute for Translational Medicine, Zhejiang University, Hangzhou, 310029 Zhejiang, China; ^3^ Key Laboratory of 3D Micro/Nano Fabrication and Characterization of Zhejiang Province, School of Engineering, Westlake University, Hangzhou, 310024 Zhejiang, China; ^4^ National Engineering Research Center for Biomaterials, Sichuan University, Chengdu 610065, China; ^5^ Clinical Research Center for Respiratory Disease, West China Hospital, Sichuan University, Chengdu 610065, China; ^6^ Institute for Advanced Study, Chengdu University, Chengdu 610106, China

## Abstract

Whether for cancer diagnosis or single-cell analysis, it remains a major challenge to isolate the target sample cells from a large background cell for high-efficiency downstream detection and analysis in an integrated chip. Therefore, in this paper, we propose a 3D-stacked multistage inertial microfluidic sorting chip for high-throughput enrichment of circulating tumor cells (CTCs) and convenient downstream analysis. In this chip, the first stage is a spiral channel with a trapezoidal cross-section, which has better separation performance than a spiral channel with a rectangular cross-section. The second and third stages adopt symmetrical square serpentine channels with different rectangular cross-section widths for further separation and enrichment of sample cells reducing the outlet flow rate for easier downstream detection and analysis. The multistage channel can separate 5 *μ*m and 15 *μ*m particles with a separation efficiency of 92.37% and purity of 98.10% at a high inlet flow rate of 1.3 mL/min. Meanwhile, it can separate tumor cells (SW480, A549, and Caki-1) from massive red blood cells (RBCs) with a separation efficiency of >80%, separation purity of >90%, and a concentration fold of ~20. The proposed work is aimed at providing a high-throughput sample processing system that can be easily integrated with flowing sample detection methods for rapid CTC analysis.

## 1. Introduction

In biomedical diagnosis, it is usually needed to separate the target samples from tissues or body fluids for high-efficiency detection and analysis. Isolated target samples usually have unique biological properties and functions, which can be used for biomedical research and clinical diagnosis, such as circulating tumor cells (CTC). CTC is considered an early marker of tumor metastasis in clinical practice and has important clinical significance in diagnosis, staging, formulation of treatment methods, and evaluation of cancer rehabilitation [1]. However, the amount of CTCs in the blood is very low, usually only 1-100 CTCs per milliliter of blood. Only by separating and enriching CTCs from the large background cells can the follow-up single-cell analysis be realized.

The separation and detection of biological samples can often rely on their physical properties such as size, deform-ability, mass, electrical properties such as membrane capacitance and cytoplasmic conductivity, or biological properties such as affinity [2]. As a CTC has an apparent size difference from other cells such as red blood cells (RBCs) or white blood cells (WBCs), they can be separated by size.

In the size-based microfluidic cell sorting technologies, dielectrophoresis (DEP) [3], acoustic sorting [4], magnetic sorting [5], optical tweezers, etc., which depend on the external force field, usually have high accuracy but low throughput and complex structures. Other technologies such as deterministic lateral displacement (DLD), pinch flow filtration (PFF) [6], and cross-flow filtration [7] can work without an external force field and have simple structures, but they are prone to blockage and need additional sheath flows. In contrast, inertial microfluidic sorting technologies only rely on the hydrodynamic force to separate particles or cells of different sizes at a high flow rate. Hence, it has great application prospects in the separation and concentration of CTCs [8].

In inertial microfluidics, the spiral channel [9–12] has attracted much attention because of its ultrahigh throughput capability. For example, Lim’s group used the Dean flow filtration (DFF) with stacked spiral channels to isolate CTC from WBC at a high throughput of 7.5 mL within 5 min [13]. Despite the fact that a high-throughput CTC separation was realized, a sheath flow was still needed which increased the complexity of the channel structure, and only a low separation purity was achieved. Then, the single inlet parallel or cascade spiral channels were proposed that realized ultrahigh-throughput and high-purity separation without sheath flow. For example, Warkiani et al. parallelized the single inlet spiral channels with trapezoidal cross-section to achieve a macroscopic volume processing rate of ~500 mL/min for CHO and yeast cell filtration [14]. And Miller et al. cascaded the single inlet spiral channels with rectangular cross-sections to improve the purity for separation and concentration of particles [15]. However, due to the limitation of the ultrahigh output flow rate, separated target cells cannot be directly detected when flowing through the channel outlet but can only be detected after the output solution was collected and transferred to a microscopic platform. This undoubtedly impedes the automation of sample preparation and detection and increases the detection complexity and the possibility of cross-contamination.

As another type of inertial microfluidics, the serpentine channel [16–18] also can sort cells of different sizes under a relatively lower flow rate compared to that of the spiral channel [19, 20]. Moreover, it can focus the target cells at the center and output the waste solution through the side outlets, hence effectively reducing the flow rate of the center collection outlet. Thus, serpentine channels are easier to be integrated with downstream detection methods. For example, Tang et al. used an asymmetric serpentine channel for impedance detection to discriminate CTCs from blood cells [21]. And Abdulla et al. combined a serpentine channel and membrane filter to sort cells for downstream single-cell analysis [22].

However, towards practical application, high flow rate input for high-throughput and low flow rate output for easy downstream detection are usually simultaneously required. Therefore, the multistage integration of the spiral and serpentine channels could potentially meet these needs, since it can not only improve the separation purity but also reduce the output flow rate, which is convenient for detection and downstream analysis [23, 24].

**Figure 1:**
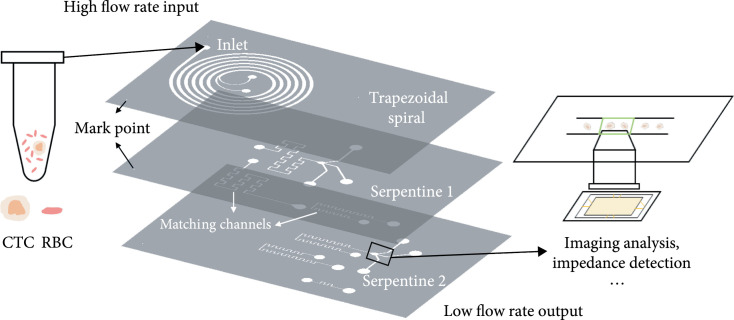
3D-stacked multistage inertial microfluidic cell sorting chip. First stage trapezoidal spiral channel at the top, second stage serpentine channel in the middle, and the third stage serpentine channel at the bottom.

Here, we propose a 3D-stacked multistage inertial microfluidic sorting chip for the enrichment of CTCs and downstream analysis. We used a trapezoidal spiral channel for the separation of CTCs and background RBCs as the first stage at the top, followed by a two-stage square serpentine channel for further removal of RBCs and purification of the target sample solution in the middle and at the bottom, as shown in Figure 1. Through the flow rate reduction with the integration of spiral and serpentine channels, a variety of detection methods such as impedance detection [25] and imaging [26, 27] can be applied at the channel collection outlet. Therefore, the multistage sorting chip can realize high flow rate input and low flow rate output and meet the requirements of medical diagnosis for throughput and detection. Furthermore, the 3D-stacked structure cast by polydimethylsiloxane (PDMS) reduces the chip areas (2 cm × 3 cm) and has good transparency for multilayer observation. The demonstrated 3D-stacked chip can separate SW480 (human colon cancer cell), A549 (human lung adenocarcinoma cell), and Caki-1 (human renal clear cell carcinoma cell) from massive RBCs at a high flow rate of 1.3 mL/min, with the separation efficiency of >80%, the separation purity of >90%, and the concentration fold of ~20. The work is aimed at providing a sample processing method that can be integrated with detection methods for rapid medical diagnosis.

## 2. Materials and Methods

### 2.1. Working Principle

Inertial microfluidics relies on inertial focusing and the Dean flow to achieve particle/cell focusing and sorting. In a simple straight channel, particles and cells focusing on the equilibrium position are only subjected to inertial lift force (ignoring buoyancy, gravity, and Brownian motion) [28]:

(1)FL=flRc,xρUma4Dh2,

where ρ is the fluid density, $U_{m}$ is the maximum rate of fluid flow ($U_{m}=2U_{f}$, $U_{f}$ is the average velocity), $D_{h}$ is the hydraulic diameter of the microchannel ($D_{h}=4A/p$, A is the area of the cross-section of the microchannel, p is the perimeter, $D_{h}\approx H$ is the minimum size of the microchannel in low aspect ratio), a is the particle size, $f_{l}\left(R_{c},x\right)$ is the lift coefficient, and its value depends on the position x of the particles in the microchannel and the microchannel Reynolds number $R_{c}$ ($R_{c}=\rho UmD_{h}/\mu$, μ is the dynamic viscosity of the fluid). But it takes a long time for the particle to move to the equilibrium position with only FL, and the equilibrium positions of different particles are too close to be separated. Therefore, the Dean flow is introduced by increasing curvature, such as spiral structure, serpentine structure, and obstacle array. When particles and cells are in the Dean flow, they will follow the Dean drag force [28]:

(2)FD=3πμUDa,

where $U_{D}\approx 1.8\times 10^{-4}\;De^{1.63}$ is the average velocity of the Dean flow and $D_{e}=R_{c}\;{\left(D_{h}/2R\right)}^{1/2}$, where R is the radius of curvature.

Under the joint action of the Dean drag force and inertial lift force, particles/cells will move to the equilibrium position quickly. Particles or cells of different sizes can be separated by different equilibrium positions. When the shape of the channel section is further changed, such as the trapezoidal section [29] and stair-like section [11], the shape of the Dean flow is changed accordingly to improve the separation performance. When the channel structure is determined, according to (1) and (2), the magnitudes of the *F_D_*and *F_L_* depend on size and flow rate. And the equilibrium position of the sample is determined by the equilibrium of *F_D_*and *F_L_* [30]. Therefore, the samples with different sizes will be separated at an appropriate flow rate. As there are obvious size differences between CTC and RBCs, they can be separated by the inertial principle.

**Figure 2:**
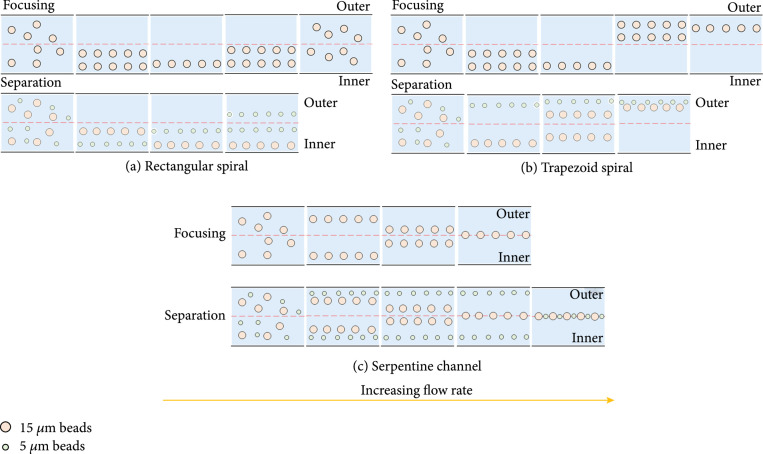
Separation principle of spiral and serpentine channels. (a) Pattern for focusing and separation of the spiral channel with rectangular section. (b) Pattern for focusing and separation of the spiral channel with trapezoidal section. (c) Pattern for focusing and separation of the serpentine channel with rectangular section.

The separation performances of the spiral channels and serpentine channels with different structures were firstly explored. As shown in Figures 2(a)–2(c), the patterns of focusing and sorting of the spiral and serpentine channel were extracted from the fluorescence image superposition method to be described in the *Experimental Setup and Data Analysis*, which is consistent with the previous studies [31–33]. The rectangular cross-section spiral channel separates cells of different sizes based on different equilibrium positions, and the trapezoidal spiral channel realizes separation depending on different flow rates for cells of different sizes jumping to the outer wall, as shown in Figure 2(b). From Figures 2(a) and 2(b), it can be seen that the trapezoidal spiral channel has a better separation distance than the rectangular spiral channel in sorting cells of different sizes, so better sorting performance can be obtained. For the serpentine channel, as shown in Figure 2(c), the larger the size of the cells, the lower the flow rate is required to focus in the center of the channel to achieve the separation of different-sized cells. As described in previous work [34], a critical threshold velocity for inertial focusing scales is $\sim 1/D_{h}$. Therefore, when the channel height remains the same and the width decreases, the required flow rate also decreases, which is also confirmed by our experiments. The multistage serpentine channel with width gradient descent can not only separate cells of different sizes but also be easier to integrate with subsequent downstream analysis and detection methods through flow rate reduction and concentration.

### 2.2. Device Design and Fabrication

**Figure 3:**
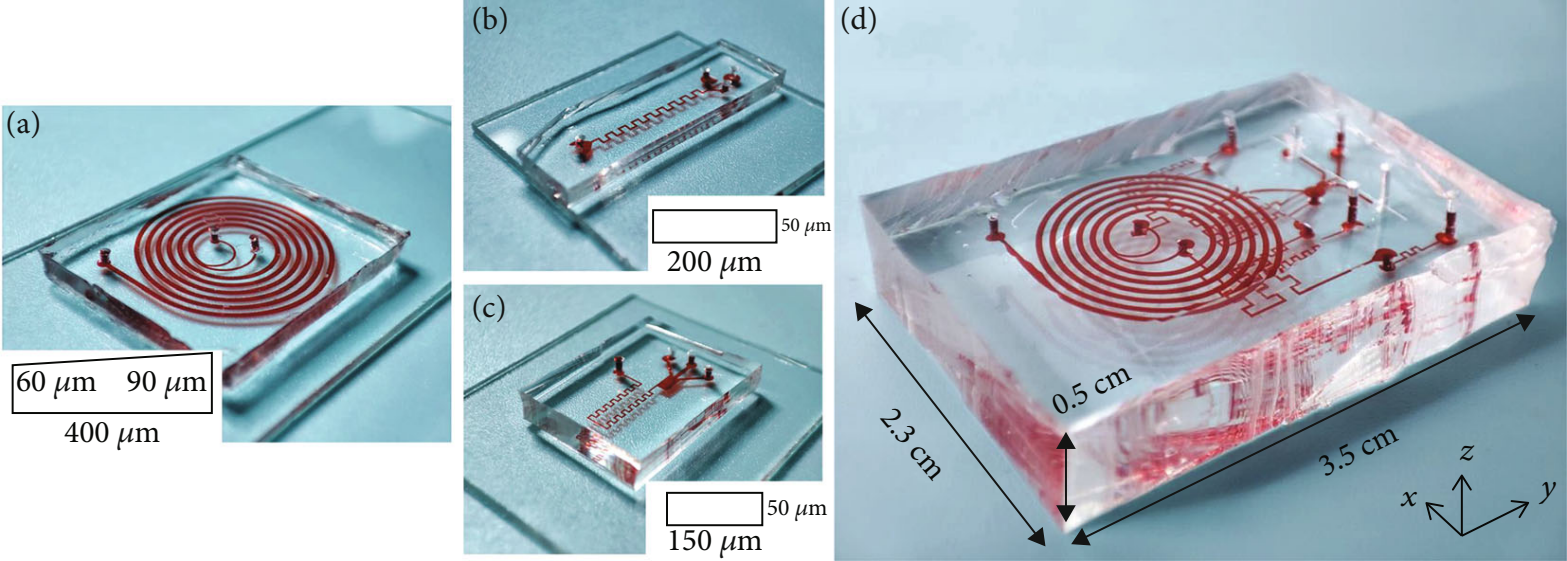
Channel structures for every stage. (a) Spiral channel with trapezoidal cross-section for the first stage. (b) A square serpentine channel ($W\times H\times L_{u}$: 200 μm×50 μm×900 μm, and the number of units is 9) for the second stage. (c) A square serpentine channel ($W\times H\times L_{u}$: 150 μm×50 μm×600 μm, and the number of units is 11) for the second stage. (d) 3D-stacked multistage chip (area: 2.3 cm×3.5 cm).

The proposed 3D-stacked multistage inertial microfluidic cell sorting chip integrated a trapezoidal spiral channel and two square serpentine channels. The trapezoidal spiral channel is the first stage of the chip with one inlet and two outlets as shown in Figure 3(a). To satisfy the focusing criterion in the trapezoidal channel ($D_{\text{outer}}/D_{\text{inner}}\geq 1.5$ and $a/D_{\text{inner}}\geq 0.07$) [29, 31, 34], the depths of the inner wall ($D_{\text{inner}}$) and outer wall ($D_{\text{outer}}$) are selected as 60 *μ*m and 90 *μ*m. The channel width (W) is 400 *μ*m to achieve a throughput>mL/min. In the trapezoidal spiral channel, with a relatively high flow rate, RBCs will focus near the outer wall, while CTC will focus near the inner wall, which leads to the separation of CTCs from RBCs.

To satisfy the focusing criterion in the serpentine channel ($a/D_{h}\geq 0.07$) and easier manufacturing, the height of the square serpentine channels is unified as 50 *μ*m. Because the trapezoidal channel outlet is divided equally, its outlet flow rate drops to about 1/2. To match the flow rate, a serpentine channel with a width of 200 *μ*m was used. Later, to further reduce the flow rate, we tested the effect of flow rate and width on separation for 150 and 100 *μ*m width serpentine channels, respectively. Finally, a square serpentine channel (Serpentine1) was adopted as the second stage with one inlet and three outlets as shown in Figure 3(b) to further deplete RBCs. The height (H), weight (W), length of each unit ($L_{u}$), and the number of units were 50 *μ*m, 200 *μ*m, 900 *μ*m, and 9, respectively. Furthermore, to slow down and purify the target flow and facilitate integrated detection methods, another square serpentine channel (Serpentine2) with one inlet and three outlets is used as the third stage as shown in Figure 3(c). The height (H), weight (W), length of each unit ($L_{u}$), and the number of units were 50 *μ*m, 150 *μ*m, 600 *μ*m, and 11, respectively.

The three stages of channels were used to design a 3D-stacked structure. The channels of different stages were arranged from top to bottom in line with the liquid flow direction with only one inlet for the whole chip without sheath flow. Moreover, to guarantee that all stages of channels could work in optimal working states, matching channels were also designed to match the flow resistance as shown in Figures 1 and 3(d). After automatic multistage removal of RBCs and the deceleration, aggregation, and concentration of target flow, the flow rate was reduced from mL/min at the inlet to *μ*L/min at the outlet, which made it easier for integration with downstream detection and analysis such as impedance detection and imaging analysis.

The whole device was cast with PDMS, while the molds of the spiral channel and serpentine channels were made in different manufacturing processes. The mold of the trapezoidal spiral channel was fabricated using a precise 3D printing technology with a printing accuracy of 2 *μ*m. For the square serpentine channel, the mold was fabricated by standard soft-lithography technique. For PDMS casting, a 10 : 1 mixed solution of base and curing agent was poured into the mold. Then, the mold was put in a vacuum drying oven to remove bubbles. After degassing, the mold was baked for 3 h at 65°C to get the cured PDMS replica. A puncher was used to make holes for liquid passage. Next, the replica was bonded to a clean glass slide or a PDMS film using a plasma machine for 20-60 s at 100 W. Finally, the bonded device was put on the heating platform at 80-90°C to further stabilize the bonding [35].

### 2.3. Sample Preparation

For the verification experiments of fluorescent particles, 5 *μ*m and 15 *μ*m fluorescent polystyrene microspheres (Baseline ChromTech Research Center, Tianjin, China) were used. The mass fraction was 100 mg/10 mL. Through calculation, the density of the polystyrene particles was 1.05 g/cm^3^, so the concentration of 5 *μ*m particles was $1.45\times 10^{8}$ particles/mL, and the 15 *μ*m particle concentration was $5.39\times 10^{6}$ particles/mL. To explore the effect of concentration on separation purity and separation efficiency, particle solutions diluted by ×50, ×100, ×200, ×400, and ×800 were prepared, respectively. The dilution reagent was mainly a phosphate buffer solution (Sangon Biotech Co., Shanghai, China), which contains 0.1% Tween20 that could prevent particles from sticking to each other.

For cell experiments, the tumor cells to be detected were SW480, A549, and Caki-1. They were cultured using Dulbecco’s modified Eagle’s medium (Gibco, China), supplemented with 10% fetal bovine serum (Gibco, Australia) and 1% penicillin/streptomycin (Biological Industries, Israel). The tumor cell lines were cultured in glass-bottom dishes with 5% CO2 at 37°C, until cell confluence reached 50-70% approximately. For background RBCs, they were obtained from peripheral blood that was collected from healthy volunteers from the Institute of Translational Medicine, Zhejiang University. Considering that the concentration of CTCs is very small, we prepared the mixed cell solution of RBC:CTC=1000:1, and the total cell concentration was about 10^6^ cells/mL diluted by PBS (excluding Tween20), which not only ensured large background cells but also facilitated the convenient observation and image recording of subsequent experiments.

### 2.4. Experimental Setup and Data Analysis

For experiments, the prepared sample solution was pumped into the developed microfluidic chip using a single channel syringe pump (WH-SP-01, Wenhao Co. Ltd., Suzhou, China) connected with a polytetrafluoroethylene (PTFE) tubing with an outer diameter of 1 mm. The waste and collection outlets were connected with 50 mL and 5 mL centrifugal tubes, respectively. In the verification experiments based on fluorescent particles, the experimental phenomena were observed and recorded by a fluorescent microscope (Olympus BX51, Tokyo, Japan) and a CCD camera and saved on a PC, which was then used to analyze the performance of the microfluidic chip at all stages.

To evaluate the separation performance of the proposed chips, parameters of separation throughput, purity, and efficiency were employed. Separation throughput refers to the sample processing speed of a microfluidic chip, which is mainly characterized by inlet flow rate or sample processing speed. Separation purity refers to the proportion of target samples in the collected samples, which is defined as the number of target samples in the collection outlet divided by the number of all samples in the collection outlet, as in (3). Separation efficiency refers to the ability to separate the target sample from the mixed solution, which is defined as the number of target samples in the collection outlet divided by the number of target samples in the inlet, as in (4).

(3)Separation purity=target samplestarget samples+nontarget samplesin collection,(4)Separation efficiency=target samplesin collectiontarget samplesin inlet.

The calculation of separation purity and efficiency requires measuring the number of target samples and nontarget samples in the solution at the collection outlet and the number of target samples in the same volume solution at the waste outlet. Therefore, we used the hemocytometers to count particles or cells 10 times to obtain the mean value and took the standard deviation as the error.

To accurately characterize these performances, it is necessary to first obtain or verify the optimal conditions through experimental data processing methods, that is, to obtain the optimal performance under the condition of the optimal flow rate and the optimal concentration. Here, fluorescent polystyrene particles of different sizes were used as the verification samples. The fluorescent trajectory images at the outlet bifurcation were captured by a fluorescent microscope. Multiple images at the same flow rate were vertically stacked by the ImageJ software. And the stacked images at different flows were montaged to obtain the fluorescent trajectory of one type of particle at different flow rates. According to the stack of different particle fluorescent trajectories, we could obtain the optimal flow rate region of particle separation. After getting the optimal flow rates, we could further explore the optimal concentration of separation through particle solutions with different dilution ratios at this flow rate. With the optimal flow rate and optimal concentration, high-efficiency separation could be realized.

## 3. Results

**Figure 4:**
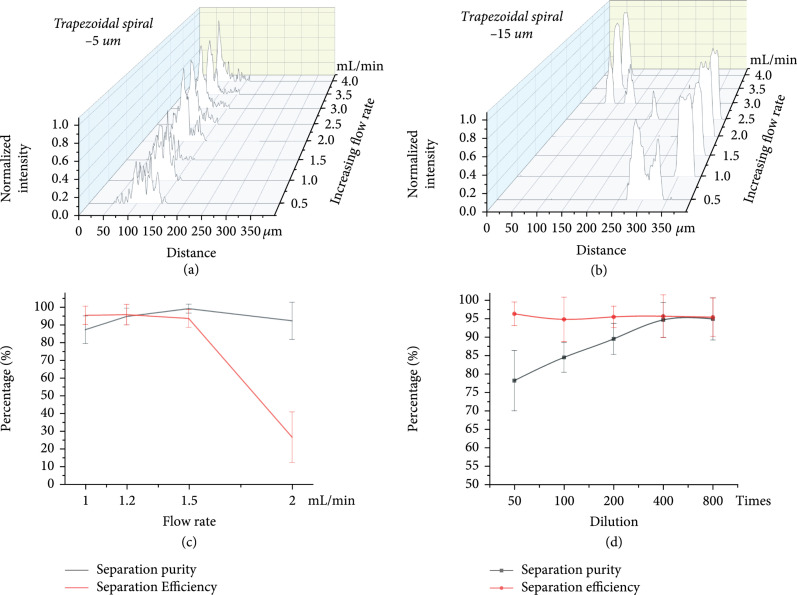
Investigate the optimal flow rate and optimal concentration for separation in the trapezoidal channel and the trapezoidal spiral channel. (a) The variation of focusing position and normalized fluorescence intensity of 5 *μ*m with flow rate. (b) The variation of focusing position and normalized fluorescence intensity of 15 *μ*m with flow rate. (c) Effect of flow rate on separation efficiency and purity. (d) Effect of solution concentration on separation efficiency and purity.

To determine the conditions under which our device can achieve the optimal separation performance, we used the first stage trapezoidal spiral channel to explore the optimal initial flow rate and concentration. The variation of focusing position and normalized fluorescence intensity of 5 *μ*m and 15 *μ*m particles with a flow rate in the trapezoidal channel is shown in Figures 4(a) and 4(b). In the flow rate region from 0.5 mL/min to 4 mL/min, the 5 *μ*m particles were focused on the outside of the channel (0-200 for the outer half of the channel). While 15 *μ*m particles were better aggregated to the inside of the channel in the flow rate region from 1 mL/min to 2 mL/min. Thus, we deduce that the optimal flow rate region for separation is 1-2 mL/min. Further, to obtain a more accurate optimal flow rate, the separation purity and separation efficiency in this flow rate region were then measured and calculated, as shown in Figure 4(c). In this region, the separation purity increases with increasing flow rate, while the separation efficiency decreases with increasing flow rate. For the trapezoidal channel, the flow rate needs to be near the intersection point in the figure to achieve high purity and high efficiency. Therefore, we chose the flow rate point near the intersection point of 1.2 mL/min as the optimal initial flow rate. In this flow rate, the separation purity reached 94.74%, and the separation efficiency reached 95.75%.

The influence of particle or cell concentration on cell sorting with the optimal initial flow rate was then explored. 5 *μ*m and 15 *μ*m mixed solution of polystyrene particles with dilutions of ×50, ×100, ×200, ×400, and ×800 was used. The results of separation purity and separation efficiency with dilution ratio are shown in Figure 4(b). With the increase in dilution ratio, the separation purity of large particles gradually increased as the interaction forces between particles were large at high concentrations but negligible at low concentrations. As can be seen from Figure 4(b), when the dilution ratio was greater than or equal to ×400, the separation purity of large particles stayed unchanged. The separation efficiency of large particles did not change much with the change in dilution ratio. To obtain as many large particles (i.e., target samples) as possible at the collection outlet, the dilution rate of ×400 for mixed particle solution with a concentration of ~10^6^ cells/mL was selected as the optimal concentration for particle separation.

After obtaining the optimal initial flow rate of 1.2 mL/min and concentration of 10^6^ cells/mL, serpentine channels of different widths and how to achieve multistage connections were explored. We used the same method as the analysis of the trapezoidal channel to obtain the flow rate region for sample separation in square serpentine channels with different widths, 500-800 *μ*L/min for the serpentine channel with 200 *μ*m width, 150-300 *μ*L/min for the serpentine channel with 150 *μ*m width, and 150-180 *μ*L/min for the serpentine channel with 100 *μ*m width. Because the outlet of the trapezoidal channel is bisected, its outlet flow rate is about 540 *μ*L/min, which is exactly in the flow rate region for sample separation of the serpentine channel with 200 *μ*m width; and the outlet of the second stage serpentine channel is divided into three equal parts, and its flow rate is also located in the region for sample separation of the serpentine channel with 150 *μ*m width. However, the inlet flow rate of the 100 *μ*m-wide serpentine channel cannot match the outlet flow rate of the 150 *μ*m-wide serpentine channel, so we do not consider it for the time being. For the channels that have achieved matching flow rates at each stage, we designed flow resistance matching channels to ensure that the inlet flow rate at each stage is within the optimal flow rate region for sample separation.

**Figure 5:**
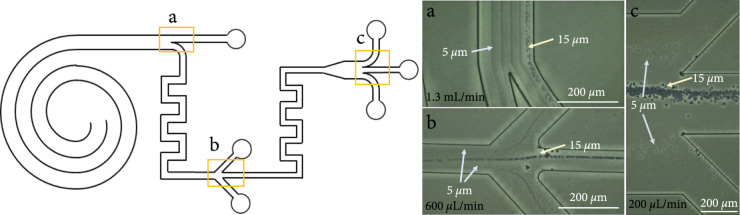
Trajectories of 5 *μ*m and 15 *μ*m particles at the outlet of each stage. (a) 15 *μ*m particles focus near the inner wall in the bifurcation of the first stage trapezoidal spiral channel with a flow rate of 1.3 mL/min. (b) 15 *μ*m particles focus in the center of the second stage serpentine channel and 5 *μ*m particles focus at the sides with a flow rate of 600 *μ*L/min. (c) 15 *μ*m particles focus near the inner wall in the bifurcation of the first stage trapezoidal spiral channel and 5 *μ*m particles focus at the sides with a flow rate of 200 *μ*L/min.

Then, experiments based on particles were further carried out to verify the designed multistage channel. With only one single channel syringe pump driving the input sample, the sorting processes at the outlets of each stage of the channel were captured by an optical microscope. The exposure time and frame rate of the microscope camera were 0.02 ms and 60 frames per second, respectively, and 10 seconds of video data were taken as the original data for image superposition in each test. Then, the particle trajectory was obtained by minimizing the vertical stacking through the ImageJ software for 600 pictures, as shown in Figure 5. It is worth noting that the experimental results may often have a certain deviation from the original design [36]. Through experiments, it was verified that the optimal input flow rate of the multistage channel was adjusted to 1.3 mL/min.

It can be seen from Figure 5(a) that a blurry streamline of 15 *μ*m particles was focused near the inner wall of the trapezoidal spiral channel, while the flow rate was too high to get the 5 *μ*m particle trajectories near the outer wall. Figure 5(b) showed the outlet of the second stage square serpentine channel. The streamline of small particles on both sides and the streamline of large particles in the channel center could be observed as the flow rate decreased, and the solution was purified after the separation and focusing of the first and second stages. Figure 5(c) shows the third stage square serpentine channel at the outlet, it could be seen that a large number of 15 *μ*m particles were focused in the center of the channel, and only a small amount of 5 *μ*m particles flew out of the waste side outlets. At the outlet of the third stage, the outline of particles could be obtained which showed that the purification and deceleration of the multistage channel could meet the design requirements.

Furthermore, we measured and calculated the separation purity and separation efficiency from the sample and waste solution collected from the outlets of all stages of the channel for accurate performance characterization. It can be concluded from Table 1 that the separation purity and efficiency after the first stage channel were 94.74% and 95.75%, respectively. After the second stage channel, the separation purity was 97.33%, and the separation efficiency was 92.80%. Finally, the separation purity was 98.10%, and the separation efficiency was 92.37% at the outlet of the multistage channel. From the results, it can be seen that the separation purity has further increased after the multistage channel, while the separation efficiency has decreased, which is also the intrinsic drawback of the multistage channel. In general, the multistage channel showed good separation performance and throughput in the particle experiments.

**Table 1:** Separation performances of channel outlets at all stages.

Channel structure	Flow rate (*μ*L/min)	Separation efficiency	Separation purity
Trapezoidal spiral	1300	95.75%	94.74%
Trapezoidal spiral + Serpentine1	600	92.80%	97.33%
Trapezoidal spiral + Serpentine1 + Serpentine2	200	92.37%	98.10%

**Figure 6:**
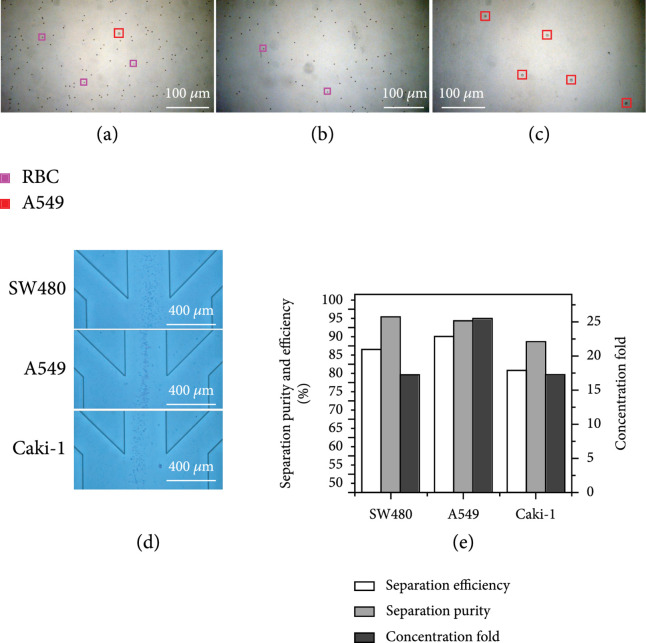
Validation of the chip using CTCs. (a) The initial mixed solution of RBCs and A549 cells under ×10 objective lens. (b) Waste solution under ×10 objective lens. (c) Collect solution under ×10 objective lens. (d) The superposition trajectories of SW480, A549, and Caki-1 at the final outlet. (e) The separation efficiency, separation purity, and concentration fold of three types of CTCs.

After the validation of the chip using particles, we used tumor cells (SW180, A549, and Caki-1) for further validation. Considering that the concentration of CTC is very small and inconvenient for observation, we prepared the mixed cell solution of RBCs:CTC=1000:1, and the total cell concentration is about 10^6^ cells/mL, which not only ensures the large background cells but also facilitates the observation and recording of subsequent experiments. As shown in Figure 6(a), take the A549 cell sorting solution as an example, in which the red box marks A549, and the purple box marks RBCs (only a small number of RBCs are marked here). It can be seen that they have large size differences, and there are few A549 cells in the prepared mixed solution. Based on the flow rate conditions used for particle verification and the fabrication of devices, we can collect the purified A549 cell solution and the RBCs solution to be removed from the collection outlet and the total waste liquid outlet, respectively, as shown in Figures 6(b) and 6(c). We removed most RBCs and obtained the A549 cell solution with high purity and increased concentration.

The separation results for SW480, A549, and Caki-1 tumor cells are shown in Figure 6(d). Stacked images with clear cell trajectory were obtained at the final outlet bifurcation with a low flow speed of about 30 mm/s. At that speed, downstream detection and analysis can be easily applied. However, the focusing positions and degrees for different cells were different, which was due to the size differences between Caki-1 (12-15 *μ*m), SW480 (12-18 *μ*m), and A549 (14-18 *μ*m). Among them, A549 cells performed best by focusing at the center of the outlet, Caki-1 cells were focused near the left wall, and SW480 cells had a larger focusing width. Furthermore, at the flow rate of 1.3 mL/min, the separation efficiency, separation purity, and concentration fold for SW480, A549, and Caki-1 cells were 89.05%, 92.59%, and 83.33%; 97.93%, 96.95%, and 91.19%; and 17, 25, and 17, respectively, as shown in Figure 6(e). At a flow rate of 1.3 mL/min, the separation efficiency was >80%, the separation purity was >90%, and the concentration fold could reach about 20. Therefore, the results of the experiments on a variety of CTCs proved that the multistage microfluidic chip had good consistency in cell sorting and could realize the sorting of multiple types of tumor cells.

## 4. Discussion

In this paper, we propose a 3D-stacked multistage inertial microfluidic chip for the enrichment of CTCs, which realized the separation of multiple types of tumor cells (i.e., SW480, A549, and Caki-1) from massive background cells with a separation efficiency>80% and separation efficiency>90%. We integrated the spiral and serpentine channels by employing their advantages and achieved the high-throughput, sheath-free, label-free, and high-purity separation of CTCs. The 3D-stacked chip cast by PDMS not only greatly reduces the chip area but also facilitates easy microscopic observation. The integrated multistage flow design prevents cross-contamination and reduces cell adhesion for collected output detection. The designed 3D-stacked multistage microfluidic chip overcomes the limitation between separation purity, separation efficiency, and throughput in a single-stage chip and provides a promising basis for integrated downstream detection methods through multistage flow rate reduction.

## Data Availability

The data used to support the findings of this study are available from the corresponding author upon request.
